# Metamaterial Based Ku-Band Antenna for Low Earth Orbit Nanosatellite Payload System

**DOI:** 10.3390/nano13020228

**Published:** 2023-01-04

**Authors:** Touhidul Alam, Mohammad Tariqul Islam, Mohammad Lutful Hakim, Khalid H. Alharbi, Mandeep Singh Jit Singh, Muntasir M. Sheikh, Rabah W. Aldhaheri, Md. Shabiul Islam, Mohamed S. Soliman

**Affiliations:** 1 Pusat Sains Ankasa (ANGKASA), Institut Perubahan Iklim, Universiti Kebangsaan Malaysia (UKM), Bangi 43600, Selangor, Malaysia; 2 Department of Computer Science and Engineering, International Islamic University Chittagong (IIUC), Kumira, Chattogram 4318, Bangladesh; 3 Centre for Advanced Electronic and Communication Engineering, Department of Electrical, Electronic and Systems Engineering, Faculty of Engineering and Built Environment, Universiti Kebangsaan Malaysia (UKM), Bangi 43600, Selangor, Malaysia; 4 Department of Electrical and Computer Engineering, King Abdulaziz University, P.O.Box. 80200, Jeddah 22254, Saudi Arabia; 5 Faculty of Engineering (FOE), Multimedia University, Persiaran Multimedia, Cyberjaya 63100, Selangor, Malaysia; 6 Department of Electrical Engineering, College of Engineering, Taif University, P.O. Box 11099, Taif 21944, Saudi Arabia; 7 Department of Electrical Engineering, Faculty of Energy Engineering, Aswan University, Aswan 81528, Egypt

**Keywords:** antenna, circular polarization, Ku-band, metamaterial, nanosatellite

## Abstract

The concept of nanosatellite technology becomes a viable platform for earth and space observation research to minimize cost and build time for the payload. The communication approach is the essential fundamental attribute of a satellite, of which the antenna is a crucial component for forming a communication link between the nanosatellite and the earth. The nanosatellite antenna must comply with some special requirements like compact size, lightweight, and high gain with a space-compatible structure. This paper proposes a compact metamaterial-based Ku-band antenna with circular polarization for the nanosatellite communication system. The designed antenna obtained an impedance bandwidth of 2.275 GHz with a realized gain of 6.74 dBi and 3 dB axial beamwidth of 165° at 12.10 GHz. The overall antenna size of the designed is 0.51λ × 0.51λ × 0.17λ, which is fabricated on Rogers 5880 substrate material. The antenna results performance has been examined with a 1 U nanosatellite structure and found suitable to integrate with metallic and nonmetallic surfaces of any miniature nanosatellite structure.

## 1. Introduction

With the advancement of technology, nanosatellite has drawn remarkable contemplation from space researchers due to the feasibility of payload missions within a few cubic centimeter’s size satellite structure and minimal expense. Nowadays, this type of small satellite is widely used in various sectors, like astronomy, astrobiology, earth observation, atmospheric science, ecology, meteorology, telecommunications, disasters mitigation and management, education, and training [1,2,3,4]. Smooth data communication is very crucial for nanosatellite missions, where the antenna plays a key role in establishing communication between the satellite and the earth. The nanosatellite antenna design becomes complex to antenna researchers due to the inverse proportionality relation between antenna performance and size [5,6,7]. Most nanosatellite payloads demand high gain, compact and circular polarized antenna for a smooth communication system [2]. 

Various antennae have been designed in Ku-band applications, but most have linear polarization. A linear polarized dual-band patch dipole antenna was developed in [8] for Ku and Ka-band uses, with resonant frequencies at 16.5 and 32.5 GHz. In [9], a larger size patch antenna was presented for S, C and Ku band frequencies. The antenna shows the resonant frequency at 2.4, 5.0, and 15.5 GHz frequencies with a low gain value. Different designs of Ku band antenna are presented in [10,11,12,13,14], where all the design has linear polarization and lower gain. Several researchers also focused on designing compact circular polarized (CP) high-gain antenna for small satellite communication systems [7,15,16]. In [12], a circular patch antenna was designed, which has CP, but the antenna size is 40 × 48 mm^2^. In [17], a patch antenna has been developed for Ku-band satellite application with a size of 20 × 20 mm^2^. The antenna shows circular polarization from 12.30 to 12.46 GHz. However, the gain of this antenna at this band is 1.6 dB. Similarly, Vijayvergiya et al. proposed a patch antenna with the size of 22.13 × 21.9 mm^2^, which operates at 12.2–14.5 GHz and has a linear polarized gain of 4.8 dB at 12.2 GHz [18]. In [19], a frequency selective surface (FSS) integrated patch antenna has been presented for a wide X and Ku band communication system, where the antenna can operate from 5 GHz to 24.5 GHz with the highest gain of 5.9 dB. The major limitation of this antenna is the overall size of 52.8 mm × 52.8 mm × 21.2 mm, which is not compatible with the 1 U (One unit) nanosatellite system. In [20], a printed monofilar antenna is offered for the Ku-band CubeSat application, which provides a gain of 8.5 dBi at 12.2 GHz with an overall size of 18 mm × 18 mm × 14 mm. The square cavity structure of the antenna makes complexity to mount with a 1 U nanosatellite structure, which is a major concern for nanosatellite antenna researchers. Therefore, from the study, it is shown that there is a great demand for designing a Ku-band antenna for a 1 U nanosatellite communication system to overcome the limitations of lower gain, larger size, complex structure, and circular polarization, etc. The metamaterial structure is attractive in antenna design, improving the gain and reducing interference with the satellite structure [21,22,23]. 

This paper proposes a metamaterial-inspired Ku band antenna for a 1 U nanosatellite communication system. The proposed antenna is a non-deployable planar structure with sufficient mechanical robustness to realize the maximum design flexibility of the limited volume 1 U nanosatellite structure. 

## 2. Antenna Design Methodology

**Figure 1:**
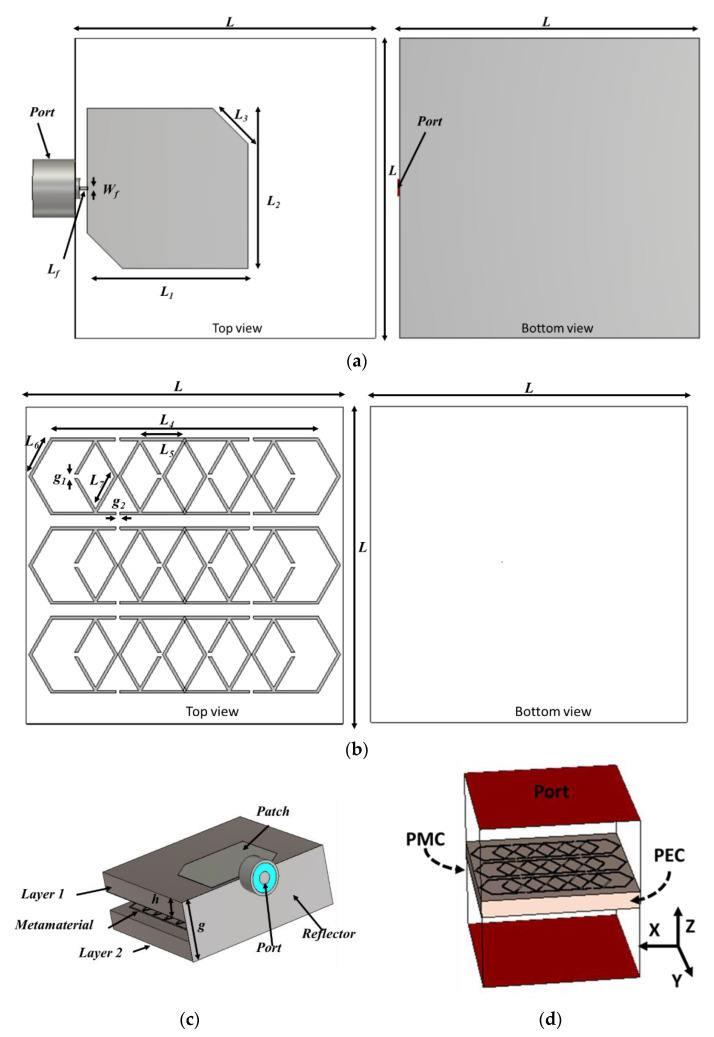
(**a**) Layer 1, (**b**) Layer 2, (**c**) perspective view and (**d**) Simulation setup of the metamaterial.

The developed antenna is intended to provide effective uplink and downlink communication between small satellites and the earth. The antenna prototype consists of two layers, shown in Figure 1. One layer is a truncated square patch antenna (Figure 1a), and another layer is an interconnected split rectangular metamaterial array (Figure 1b). Both layers are interconnected with conducting reflectors shown in Figure 1c. The space between the antenna and the metamaterial layer is 1.69 mm. Both layers are connected with a 0.25 mm thick conductive reflector wall, which also provides a strong mechanical strength of the structure. The antenna is fabricated on space-quality substrate material Rogers 5880 with a thickness of 1.575 mm, a dielectric constant of 2.2 and a dielectric loss tangent of 0.0009. The antenna is fed by a 50 Ω Sub-Miniature version A (SMA) connector. The optimized structural layout parameters are listed in Table 1. The overall size of the antenna will be 15 mm × 15 mm. Figure 1b shows the designed net-like hexagonal split ring base metamaterial structure, which has equal dimensions to the antenna and all other design parameters of the antenna listed in Table 1. The metamaterial structure has been characterized by considering perfect magnetic (PMC) and perfect electric (PEC) boundary conditions along the y and x-axis. The designed metamaterial-based antenna has been presented in Figure 1c. The designed metamaterial structure simulation setup is presented in Figure 1d [24]. 

The metamaterial property like relative permittivity (ε_r_) and permeability (µ_r_) has been calculated from scattering parameters by using Equations (1) and (2) [25,26,27].

(1) εr=2jk0d×1−S11−S211+S11+S21

(2) μr=2jk0d×1−S21+S111+S21−S11 

**Figure 2:**
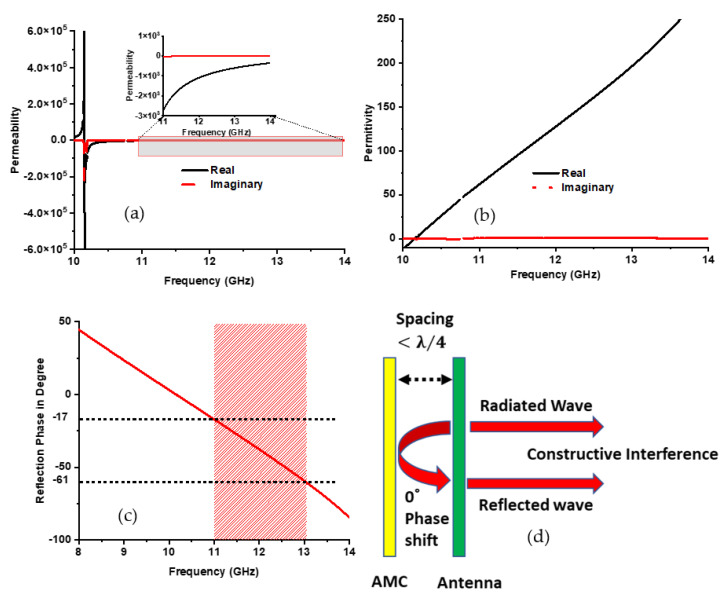
Metamaterial characteristics (**a**) permeability; (**b**) permittivity. (**c**) reflection phase, and (**d**) AMC wave interference.

Figure 2a,b shows the metamaterial property of the designed metamaterial reflector. The higher negative permeability has been achieved at the operating frequency of the antenna (10.18–13.05 GHz), which is µ_r_ ≥ −600. On the other hand, higher positive permittivity appeared in the same frequency range. This single negative feature reflected back the incident electromagnetic wave. The phase information of the reflected wave can also be understood from the phase value of the reflection coefficient. Figure 2c shows the phase value of the proposed metamaterial reflection operating frequency relayed in the +90° to −90° range. This is characterized as an artificial magnetic conductor (AMC) because the operating bandwidth of the AMC is considered from +90° to −90°, and the resonant frequency is considered at 0-degree. The AMC-type metamaterial structure mimics the attributes of the perfect magnetic conductor. The AMC, which has PMC characteristics, can reflect the incident wave with a 0-degree phase, which made a constructive interference with the antenna-radiated wave in the forward direction shown in Figure 2d. Hence, the directivity and gain of the antenna improve significantly, and interference of the signal with the back side element of the antenna has been reduced. 

## 3. Results and Discussions

**Figure 3:**
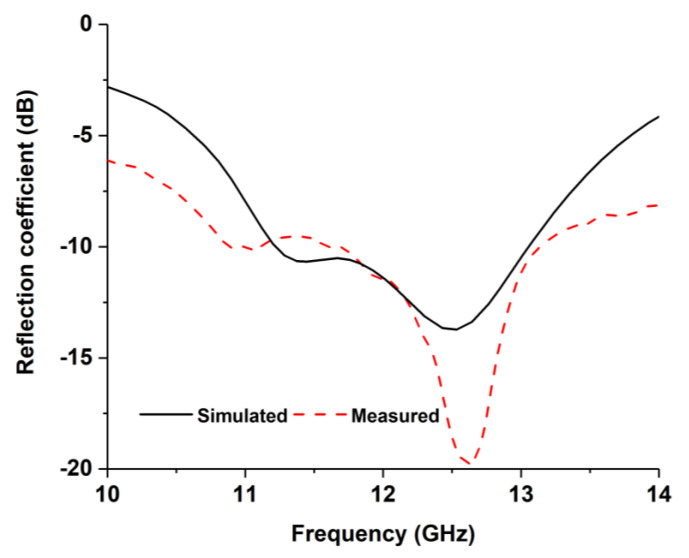
Reflection coefficient results.

The simulated antenna has been fabricated and measured to validate performances. The antenna’s reflection coefficient is presented in Figure 3. The antenna achieves −10 dB impedance bandwidth of 1.87 GHz (11.18 GHz to 13.05 GHz) in simulation and 2.275 GHz (10.85 to 13.125 GHz) in measurement. Both results seem identical. However, a little mismatch is observed due to fabrication and measurement tolerances.

**Figure 4:**
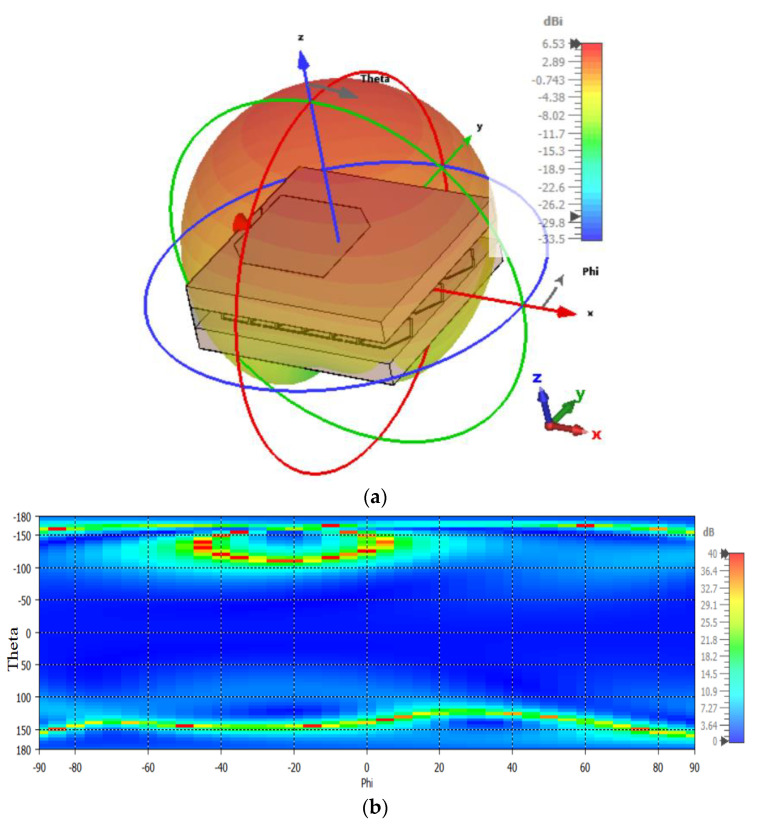
(**a**) 3D radiation pattern and (**b**) axial ratio at 12.1 GHz.

The simulated 3D radiation pattern of the projected antenna at 12.1 GHz is demonstrated in Figure 4a. The antenna shows 6.53 dBi of realized gain with very low back radiation. The substantial decrease in back radiation occurred due to the AMC metamaterial layer. Additionally, the 3 dB simulated axial ratio is also presented in Figure 4b. The antenna shows approximately 165° of 3 dB axial beamwidth with stable circular polarization.

**Figure 5:**
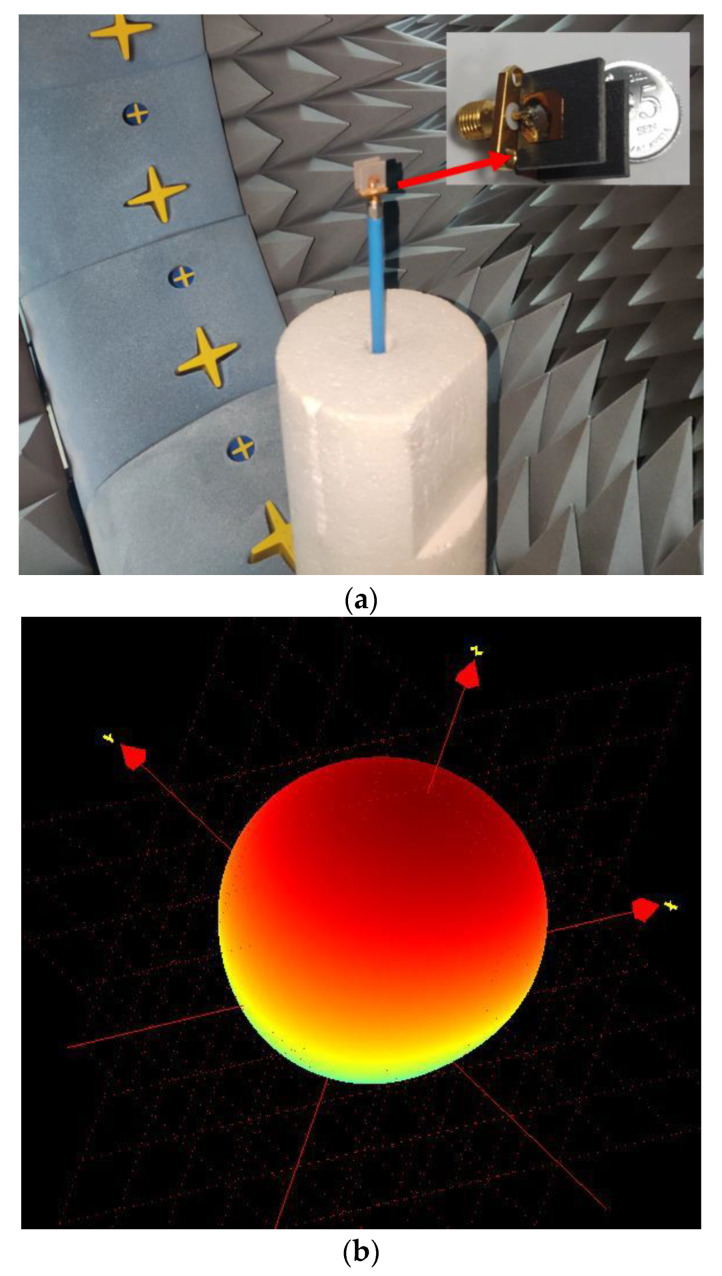
The radiation pattern of the antenna (**a**) measurement system and (**b**) measured radiation pattern.

**Figure 6:**
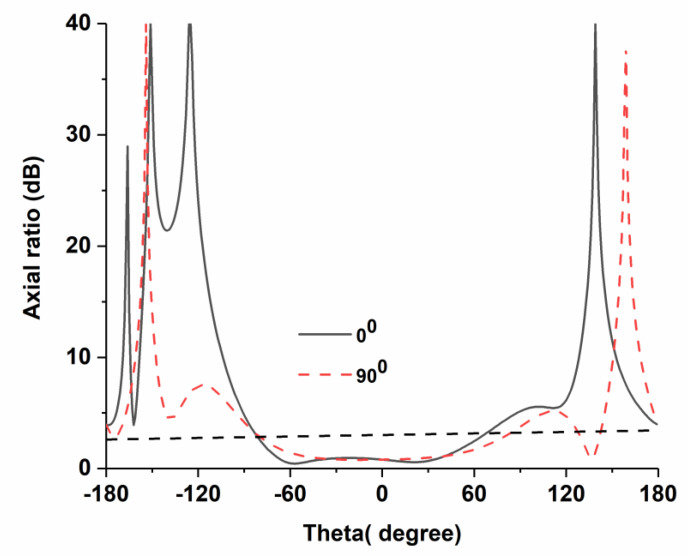
Axial beamwidth of the proposed antenna.

The radiation characteristics have been measured in Satimo nearfield measurement system, shown in Figure 5. The antenna exhibits realized gain of 6.61 dB and 6.7 dB without nanosatellite (free space) and with nanosatellite structure, respectively. Besides, axial beamwidth at 12.1 GHz has also been measured, shown in Figure 6. The antenna attained 3 dB axial beamwidth of 162° and 158° for phi 0° and phi 90°, correspondingly. 

**Figure 7:**
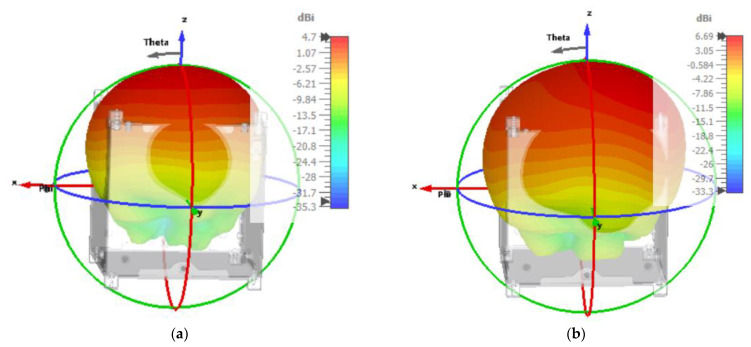
Antenna radiation pattern with 1 U nanosatellite structure at 12.1 GHz (**a**) without AMC metamaterial and (**b**) with metamaterial.

The developed antenna has been integrated into the standard 1 U nanosatellite architecture to investigate the performance in the real environment. In both simulation and measurement, the antenna reveals well agreement in both measurements. The radiation pattern with 1 U nanosatellite structure at 12.1 GHz is depicted in Figure 7. In Figure 7a, only antenna (without metamaterial layer) has been integrated, and radiation performances have been observed where the antenna reveals realized gain of 4.7 dB. On the other hand, the antenna with a metamaterial layer has been integrated to observe radiation performances, where the antenna reveals realized gain of 6.69 dB. Therefore, the metamaterial significantly improves antenna gain and reduces the coupling effect with satellite structure. 

This article presents a compact Ku-band CP antenna for the nanosatellite communication system. The antenna’s gain significantly increases by placing an AMC in the back side of the antenna, which also reduces the antenna’s signal interference with other nanosatellite components. The antenna’s performance has been investigated with a 1 U nanosatellite structure and found suitable to integrate with metallic and nonmetallic surfaces of any miniature nanosatellite structure. A details comparison table of the projected antenna with the existing antenna has been presented in Table 2. Where most of the shows liner polarization except [12], but the antenna size is larger than the projected antenna. Additionally, antenna performance does not investigate with satellite structure. The antenna presented in [28] and [11] shows higher gain, but the antenna size is very large compared to the nanosatellite structure; polarization is the liner. Finally, the compact size, high gain, circular polarization, and investigation with 1 U nanosatellite structure makes proposed antenna a potential candidate for Ku-band 1 U nanosatellite communication systems. 

## 4. Conclusions

This paper presents a non-deployable circular polarized antenna for the Ku-band 1 U nanosatellite communication system, which is highly mechanical robust with 1 U structure. The antenna prototype has been designed, fabricated, and measured. By adopting AMC metamaterial layer with reflector wall technique, the antenna achieves circular polarization with a realized gain of 6.69 dB with 1 U nanosatellite structure. Therefore, an extensive and comprehensive performance analysis of the proposed antenna shows its suitability with a nanosatellite environment.

## Figures and Tables

**Table 1:** Layout parameters of the presented antenna.

Parameters	Value (mm)	Parameters	Value (mm)
L	14.22	L7	2.0
L1	5.93	Lf	0.4
L2	5.93	Wf	0.2
L3	2.36	g1	0.2
L4	12	g2	0.2
L5	1.85	h	1.57
L6	2	g	4.83

**Table 2:** Comparison with the existing antenna.

Ref	Antenna Type	Size	Substrate	Resonant Frequency	Bandwidth (GHz)	Gain (dBi)	Polarization
[8]	Patch-dipole antenna	10 × 10 × 1.524	TSM-DS3	16.5 32.5	15.9–17.7 31.4–34.4	6.8	Linear
[9]	Circular slot rectangular patch and an inverted-L strip	40 × 40 × 1.6	FR-4	2.4 5.0 15.5	2.10–2.70 4.82–6.10 12.73–18	2.35 4.41 4.71	Linear
[29]	Circular slot patch and parasitic inverted L-shape	40 × 40 × 1.6	FR-4	2.4 5.0	2.02–2.62 5.08–6.27 11.97–18	2.27 4.02 5.05	Linear
[10]	Arrow Shaped Microstrip Patch Antenna	12 × 15 × 1.6	FR-4	17	6.75–18.96	7.41	Linear
[14]	Modified multi-slot patch		RT5880, graphene	11.46 15.42	8–18	6.98 8.14	Linear
[28]	Defected ground antenna with Microstrip-line-fed	36 × 36 × 0.762	Duroid-5880	10.8 16	9.8 to 17.55	5–12.08	Linear
[13]	Low-profile patch antenna	20 × 20 × 1.6	FR-4	12.38, 14.40	11.69–13.24 13.72–15.07	1.6–4.2	Linear
[12]	Hexa-decagon circular patch antenna with DGS.	40 × 48 × 1.59	FR4	13.67 15.28	13.179–14.033 14.584–15.724	8.01 6.01	CP
[30]	Defected ground and multiple slots antenna	20 × 20 × 1.6	FR4	N/A	11.40–12.91 13.86–14.53 17.20–17.86	2.08–6	Linear
[11]	Antenna based on single layer meta-surface	80 × 80 × 14.5	FR4	N/A	12–14.13	14.2	Linear
proposed	AMC base patch antenna	14.22 × 14.22 × 4.83	Duroid-5880	12.1	11.18–13.05	6.8	CP

## Data Availability

The data presented in this study are presented in this article.
